# Editorial: Development of stroke systems of care across the globe

**DOI:** 10.3389/fneur.2023.1292036

**Published:** 2023-09-26

**Authors:** Sheila Cristina Ouriques Martins, Thaís Leite Secchi, Carlos Molina, Raul Nogueira

**Affiliations:** ^1^Department of Neurology, Hospital de Clínicas de Porto Alegre, Universidade Federal do Rio Grande do Sul, Porto Alegre, Brazil; ^2^Department of Neurology, Hospital Moinhos de Vento, Porto Alegre, Brazil; ^3^Department of Neurology, Vall d'Hebron Hospital, Barcelona, Spain; ^4^Department of Neurology and Neurosurgery, University of Pittsburgh, Pittsburgh, PA, United States

**Keywords:** global stroke care disparities, telestroke programs, stroke care access, rehabilitation services, stroke network

Stroke, a leading cause of disability and death worldwide, knows no boundaries, affecting individuals, families, and communities globally and imposing immense challenges on healthcare systems (1). To address this, stroke care encompasses a continuum of services, ranging from prevention and acute treatment to rehabilitation and long-term support, highlighting the importance of effective stroke systems of care to ensure optimal patient outcomes.

The Research Topic “*Development of stroke systems of care across the globe*” includes 21 manuscripts: 14 original research articles, one systematic review, one study protocol, two reviews, one prospective, and two brief research reports. These articles provide valuable insights into stroke care delivery across different regions and healthcare settings. They cover early identification and assessment of stroke patients, innovative technologies for acute stroke management, the impact of telestroke programs on specialized care access, and comprehensive rehabilitation strategies.

Despite the universal impact of stroke, substantial inequalities exist between countries and regions. High-income countries often possess well-established stroke networks, specialized stroke centers, and comprehensive rehabilitation programs. In contrast, low- and middle-income countries face significant challenges in terms of limited resources, inadequate infrastructure, and a shortage of healthcare professionals (2). As an example of these inequalities in a middle-income country, the SAMBA study, an analysis of stroke in multiple Brazilian areas, addresses the significant impact of stroke in Brazil with a remarkable social and financial burden (Santos et al.). The study registered 932 stroke cases in 1 year, revealing different incidence rates among the cities. Sobral, with the lowest socioeconomic indexes and no stroke service in the city, exhibited the worst results in terms of lethality (49% in hospital mortality) and functional status. In this context, Matuja et al. conducted a study in Tanzania to assess the prevalence and outcomes of presumed large vessel occlusion (LVO) in ischemic stroke patients, with a particular focus on a region where definitive vessel imaging is not readily available. The study revealed a high burden of presumed LVO, resulting in significant 1-year morbidity and mortality rates, emphasizing the need for good epidemiological data before advocating for evidence-based acute stroke interventions in resource-limited settings. These studies highlight the importance of improved resource allocation based on regional health priorities to improve stroke care and outcomes in vulnerable populations.

Disparities in stroke care are not only limited to income levels but also emerge between rural and urban regions, as demonstrated by Llanos-Leyton et al.. In a prospective cohort study conducted in Colombia, stroke patients from diverse areas were examined, and their functional outcomes were evaluated based on sociodemographic factors and healthcare access. The study revealed that rural patients had a higher likelihood of experiencing severe strokes and unfavorable functional outcomes at discharge and during the 3-month follow-up, in contrast to urban patients with similar risk factors. Importantly, these disparities could not be solely attributed to poverty rates or barriers in healthcare access. Gender differences in stroke outcomes and treatment access also require attention. Naveed et al., using Qatar stroke database data, discovered gender disparities in stroke care. Women had delayed presentation, more severe strokes, lower thrombolysis rates, increased complications, and longer hospital stays. Long-term outcomes were unfavorable for women, with fewer achieving good outcomes at 90 days, lower medication use, and higher rates of major adverse cardiovascular events.

Yang et al. provides an essential addition to the discourse on stroke care challenges. The results gleaned from the study indicate that pre-stroke frailty is an independent risk factor for 28-day mortality and for 28-day or 1-year severe disability post-stroke. Analyzing data from international aging surveys involving 3,432 participants with stroke history, Gil-Salcedo et al. found that individuals with higher pre-stroke disability experienced less pronounced increases in limitations in activities of daily living (ADL) 1 year after the stroke. However, over time, ADL limitations increased for all pre-stroke disability levels, particularly in those aged 75 or older at stroke onset. This highlights the necessity of adopting an inclusive approach to stroke care that not only takes into account clinical variables but also considers patients' pre-existing conditions. In conjunction with the disparities mentioned earlier, these findings further accentuate the significance of implementing stroke care systems attuned to the diverse needs of populations across varying healthcare contexts.

Addressing these inequalities requires a multi-faceted approach involving targeted funding, capacity building, and knowledge transfer. A well-coordinated stroke network is essential for efficient healthcare delivery, including hospitals, emergency medical services (EMS), and rehabilitation centers. EMS utilization among acute ischemic stroke has been proven to significantly shorten prehospital delay and enhance prenotification of the receiving hospital (3). Ding K. et al. conducted an analysis of medical records from 2018 to 2021 in Beijing, uncovering that 46.1% of acute ischemic stroke patients were transported to hospitals via EMS. This study identified significant disparities in EMS usage, with a notable preference for urban areas. Nasreldein et al. investigated factors contributing to pre- and in-hospital delays in the use of intravenous thrombolysis (IVT) for acute ischemic stroke patients in both urban and rural areas of Egypt. Rural patients experienced longer delays from symptom onset to hospital arrival, emphasizing the need for improved educational initiatives and enhanced EMS accessibility, particularly in rural regions and for individuals at an elevated risk of stroke.

Accurate prehospital triage for acute ischemic stroke (AIS) patients is crucial to ensure timely and appropriate care, preventing the misallocation of resources. Sjöö et al. evaluated the characteristics and diagnosis distribution among patients presenting with suspected stroke and stroke mimics within the newly implemented Stockholm Stroke Triage System. The study showed the importance of accurately triaging stroke mimics to avoid inappropriate resource allocation. Recently, studies have shown that mobile stroke units (MSU) care expedites intravenous thrombolysis compared to standard emergency medical services. In this context, Ellens et al. conducted a study to establish standard metrics for reporting MSU operational efficiency, considering that MSU operations require significant personnel and material resources, and the cost-effectiveness and viability of these units will vary according to local circumstances.

At its core, the stroke network emphasizes swift identification and diagnosis, rapid transport of patients to designated stroke centers, and seamless communication among medical professionals. This cohesive approach ensures that stroke patients receive the most appropriate treatment promptly, leading to a higher likelihood of successful recovery (4). Bonifacio-Delgadillo et al. present a remarkable stroke network program, *the ResISSSTE Cerebro*, which was established in 2019 as Mexico's first stroke network in the public health system. This program features one advanced stroke center and seven essential stroke centers using a modified hub-and-spoke model to deliver acute stroke care and showcases positive clinical outcomes. Schaefer et al. conducted a retrospective analysis using data from the German Stroke Registry to compare outcomes between two models of endovascular therapy (EVT) for AIS patients with large-vessel occlusion: the “drip-an-ship” model and direct transfer to a thrombectomy center. Their findings indicated that secondary transfers in the DS model resulted in poorer outcomes when compared to the direct-to-center EVT approach, emphasizing the need to optimize EVT workflows to reduce time delays.

The organization of stroke services within hospitals assumes a pivotal role in optimizing patient outcomes and fostering recovery. Rapid and efficient triage, accurate diagnosis, and effective treatment pathways collectively contribute to a significant reduction in the impact of strokes, thereby enhancing survival rates. Sahakyan et al. present a contemporary evaluation of the implementation of acute stroke care services in Armenia, subsequent to the establishment of the National Stroke Program in 2019. Among the 385 patients included in the analysis, 155 underwent reperfusion therapies, primarily arriving at the hospital via ambulance. Notably, 79.2% exhibited neurological improvement upon discharge, and 60.6% achieved a favorable modified Rankin score of 0–2 at the three-month mark. The study highlights the substantial progress achieved in the performance of acute stroke services through the implementation of organized protocol-driven care, serving as a model for enhancing structured stroke care in resource-limited settings. During the COVID-19 pandemic, numerous challenges emerged in maintaining stroke patient care in hospitals, including issues related to access, infection control, and resource allocation. Klu et al. conducted a study in Brazil, demonstrating the feasibility of reducing the door-to-needle time for acute stroke patients. Importantly, their research highlights that despite the challenges posed by the pandemic, acute stroke care remained a priority. Also, the study emphasizes the continuous monitoring of service times to enhance stroke center quality and improve functional outcomes for stroke patients.

Furthermore, a well-structured system ensures the delivery of appropriate post-acute care and rehabilitation services, which are integral in reinstating functional capabilities and augmenting overall quality of life. Su et al. conducted a meta-analysis of telerehabilitation's impact on stroke patients during the pandemic, showing its effectiveness. Equally vital is the establishment of robust follow-up mechanisms for stroke patients subsequent to their initial hospitalization. Illustratively, Yi et al. examined a cohort of 2,893 individuals at high risk of stroke in China. The study revealed that over a span of 4.7 years, the rates of persistence with various medications ranged from 38.0 to 59.8%. Notably, the study observed a direct correlation between increased adherence to antihypertensives, hypoglycemics, lipid-lowering medications, and antithrombotics, and a reduced incidence of new ischemic strokes and composite vascular events. These findings underscore the imperative of augmenting the persistence of drug therapy through public education, particularly among high-risk populations. Technology plays a crucial role in identifying high-risk patients, enabling timely interventions and regular monitoring, which can significantly improve long-term health outcomes. Gong et al. demonstrate the cost-effectiveness evaluation of implementing a primary care-based and technology-enabled model of intervention, which is centered on stroke secondary prevention and management in rural China. Sustained continuity of care, facilitated by regular follow-up appointments, empowers healthcare providers to closely monitor the patient's progress and manage risk factors, ultimately culminating in the diminishment of recurrent stroke risks.

Also, effective stroke care systems require continuous learning, knowledge exchange and government support. Ding G. -B. et al. assessed stroke knowledge among primary healthcare providers in the context of acute stroke care and found that only 39.7% of healthcare providers surveyed were aware of the time window for stroke management. These findings highlight the importance of implementing strategies to enhance stroke-related knowledge and awareness of healthcare professionals. The field of stroke care has witnessed significant advancements in recent years, driven by the collective efforts of researchers, clinicians, and policymakers globally. Amorín et al. showed that Uruguay's National Stroke Plan serves as a prime example of successful collaboration, supported by financial incentives to adhere to stroke management guidelines, to improve quality of stroke care, even during the pandemic. The authors demonstrate that by focusing on these key aspects, better outcomes, improved quality of life for stroke survivors, and reduced healthcare costs can be achieved. Stroke registries have been implemented for quality improvement, resulting in an increased thrombolysis rate. Wada et al. explore the establishment and significance of the Japan Stroke Data Bank in the context of the Japanese National Plan for the Promotion of Measures Against Cerebrovascular and Cardiovascular Diseases. They emphasize the importance of improving the registry's quality through meticulous data collection, integration with external databases, enhancing treatment quality via benchmarking, and securing ongoing support from governmental and academic institutions. Collantes et al. described the gaps in stroke care and the development of stroke systems of care in the Philippines. The Stroke Society of the Philippines has collaborated with the government to address these issues by providing nationwide and regional stroke training, establishing acute stroke-ready hospitals and units, and adapting stroke protocols. Other low- and middle-income countries can learn valuable lessons from these studies by utilizing technology for outreach, training non-neurologists to assist stroke patients, expanding insurance coverage for reperfusion therapies, improving stroke infrastructures, and bolstering community awareness about stroke.

In conclusion, effective stroke systems of care require global collaboration, continuous learning, and innovative strategies tailored to diverse regional needs. Addressing disparities, promoting knowledge exchange, and implementing early interventions are essential steps in reducing the global burden of stroke and improving outcomes for patients.

## Author contributions

SM: Conceptualization, Writing—review and editing. TS: Writing—original draft, Writing—review and editing. CM: Writing—review and editing. RN: Writing—review and editing.
